# Heart rate estimation from ballistocardiogram signals processing via low-cost telemedicine architectures: a comparative performance evaluation

**DOI:** 10.3389/fdgth.2023.1222898

**Published:** 2023-07-31

**Authors:** Adriano Tramontano, Oscar Tamburis, Salvatore Cioce, Salvatore Venticinque, Mario Magliulo

**Affiliations:** ^1^ Institute of Biostructures and Bioimaging, National Research Council (IBB–CNR), Naples, Italy; ^2^ Department of Veterinary Medicine and Animal Productions, University of Naples “Federico II”, Naples, Italy; ^3^ Department of Engineering, University of Campania “Luigi Vanvitelli”, Aversa (CE), Italy

**Keywords:** eHealth, bpm, photoplethysmography, ballistocardiography, computer architecture, signal processing, neural networks

## Abstract

Medical devices (MDs) have been designed for monitoring the parameters of patients in many sectors. Nonetheless, despite being high-performing and reliable, they often turn out to be expensive and intrusive. In addition, MDs are almost exclusively used in controlled, hospital-based environments. Paving a path of technological innovation in the clinical field, a very active line of research is currently dealing with the possibility to rely on non-medical-graded low-cost devices, to develop unattended telemedicine (TM) solutions aimed at non-invasively gathering data, signals, and images. In this article, a TM solution is proposed for monitoring the heart rate (HR) of patients during sleep. A remote patient monitoring system (RPMS) featuring a smart belt equipped with pressure sensors for ballistocardiogram (BCG) signals sampling was deployed. A field trial was then conducted over a 2-month period on 24 volunteers, who also agreed to wear a finger pulse oximeter capable of producing a photoplethysmography (PPG) signal as the gold standard, to examine the feasibility of the solution via the estimation of HR values from the collected BCG signals. For this purpose, two of the highest-performing approaches for HR estimation from BCG signals, one algorithmic and the other based on a convolutional neural network (CNN), were retrieved from the literature and updated for a TM-related use case. Finally, HR estimation performances were assessed in terms of patient-wise mean absolute error (MAE). Results retrieved from the literature (controlled environment) outperformed those achieved in the experimentation (TM environment) by 29% (MAE = 4.24 vs. 5.46, algorithmic approach) and 52% (MAE = 2.32 vs. 3.54, CNN-based approach), respectively. Nonetheless, a low packet loss ratio, restrained elaboration time of the collected biomedical big data, low-cost deployment, and positive feedback from the users, demonstrate the robustness, reliability, and applicability of the proposed TM solution. In light of this, further steps will be planned to fulfill new targets, such as evaluation of respiratory rate (RR), and pattern assessment of the movement of the participants overnight.

## 1. Introduction

There is an ever-pressing need in the medical field to acquire data, signals, and images related to the health status of patients when they are not hospitalized, and therefore correlated to their instrumental activities of daily living (IADLs) (1, 2). Specific medical devices (MDs) have been designed for clinical trials in many sectors, which are capable of properly acquiring high-quality data, yet quite often such devices are both expensive and intrusive (3–5). Finding new ways to achieve comparable results, therefore, becomes critical. In this regard, a promising way focuses on reengineering low-cost, non-MD technologies already available on the market. These make it possible to realize non-invasive patient parameter monitoring systems, although they are characterized by lower performance than that achieved by systems embedding MDs (6, 7).

A recent trend focuses on sleep-related diseases — such as sleep-disordered breathing (SDB), better known as obstructive sleep apnea syndrome (OSA) — and associated cardiovascular complications, which are among the most common clinical sleep disorders (8). Polysomnography is the gold-standard approach to accurately diagnose OSA, but MDs currently deployed to monitor the parameters of patients during sleep are among the most expensive and intrusive. This means that in a hospital-based, controlled environment (i) monitoring activities are only feasible for a few patients at a time (or even for one single patient) and (ii) monitoring periods usually do not exceed one single night. Further, the results of observations may be affected by inaccuracy induced by the intrusiveness of the monitoring device (9–11).

Based on these premises, ballistocardiography (BCG) is a viable, cost-effective approach that allows the measurement of body movements and small vibrations coming from various sources, including respiration and the contractions of the heart (12). Thanks to both recent advancements in terms of vibration sensing technology and the unobtrusiveness of the sensing devices, a remote patient monitoring system (RPMS) making use of BCG signals can be deployed in different kinds of environments and seamlessly collect biomedical parameters, without burdening patients and clinical personnel. As the interest in the impact of RPMSs on current eHealth dynamics is rapidly increasing (13), the principle according to which well-gathered data can become well- and easily-processed data, once working in real/realistic remote monitoring contexts, likely demands seeking solutions relying on different paradigms to be proved.

The present article aims at figuring out and deploying a telemedicine (TM) solution for a non-intrusive, continuous remote monitoring of the status of patients during sleep, which features the following fundamental aspects:

• an unattended RPMS able to collect continuous BCG signals for heart rate (HR) estimation;
• use of unobtrusive sampling devices that can be well-tolerated by the monitored patients, so as to minimize the impact on measurement accuracy and allow a better integration with patients' IADLs;
• low costs for set up and implementation, so as to spread it out to wider cohorts of patients.

A field trial was conducted in the period between September and November 2022 in a cohort of 24 volunteers differing in gender, weight, and age who were asked to place under their bed sheets, during sleeping hours, a band equipped with pressure sensors suitable for sampling BCG signals. A dedicated prototype RPMS architecture, based on machine-to-machine (M2M) communication (14), was then designed for remote gathering of the BCG signals. Different, well-acknowledged methodologies to extract the heart rate (HR) from the BCG signals from data not gathered via RPMSs have been developed in the literature [see e.g. (15–17),]. In line with this, Pröll proposed two approaches for HR estimation, one algorithm-based (18) and one deploying deep learning networks (19): in both cases, they proved their approach outperforms those coming from the previous contributions. Therefore, recognizing Pröll's work as the field's state of the art, the authors tried to reinterpret the two mentioned approaches for a TM-related, non-controlled environment. For each of them, HR was extracted from the BCG signals, and the mean absolute error (MAE) was calculated. The results were then compared with the errors retrieved from the literature so as to validate the overall robustness and feasibility of the proposed TM solution.

The article is articulated as follows: after the introduction, an analysis of the literature is reported concerning either heart rate estimation from BCG signals or the architectures for remote monitoring of vital signs. The prototype architecture, along with the field trial conducted (including the main results), are described. The discussion and conclusions are then shown in the final sections.

## 2. Related work

The study of the literature mainly focused on the following two aspects: the evolution of signal processing techniques for HR estimation from BCG; and trend analysis of telemedicine for measuring biomedical parameters.

### 2.1. Heart rate estimation from BCG

Ballistocardiography, as the measurement of the body's micromovements due to blood ejected from the heart and moved in the large vessels, is a promising alternative for pulse rate and heart rate variability (HRV) assessment (12–20). Current technologies such as electrocardiography (ECG) or echocardiography proved in the past to be more reliable in clinical practice because of better reproducibility and fewer inter-individual differences. On the other hand, they also turned out to be uncomfortable for the patients, as either skin-electrode interfaces or flexible bandages could cause skin irritations after long use (21). BCG is instead causing increasing research interest mostly due to the advancement of vibration sensing technology, improved unobtrusiveness of the sensing devices, and increased computational power, which provides clear advantages in terms of ease of use, comfort level, and cost, thus allowing for wide applications at homes or hospitals (18–22). The presence of strong variation for what concerns the morphology of individual heartbeats still turns out to be a key challenge for a correct analysis of BCG. Causes are manifold and related to, for example, the position and the posture of the subject in the bed during sleep time. This makes it quite difficult to get to a noiseless assessment of the beat-to-beat interval, or HR, over time. In addition, BCG is a nonstationary signal. This means that, although the HR evaluation from BCG in the frequency domain does not rely on the presence of specific peaks or templates within the waveform, in a fixed BCG signal window, a prominent frequency component may result anyway, missing most of the time (23, 24). To overcome such a shortcoming, a number of related peak detection and/or heartbeat interval estimation methods are proposed in the literature. In Suliman et al. (22), a “multi-method” algorithm framework was described, which was capable of comparing five different BCG peak detection methods. Three of these were recreated from the literature (16, 25, 26), whereas the remainder were adapted from codes originally provided by Lydon et al. (27) and Sadek et al. (28) to address differences in sensing methods and sampling frequencies. A promising method named HTPV was described by (29), which combines the Hilbert transform (HT) and a phase vocoder (PV) for heart rate estimation from the frequency domain, so as to avoid heartbeat detection. Issues emerged in cases with motion artifacts caused by body movement. Instead, following the original empirical mode decomposition approach postulated for the first time by Huang et al. (30) to estimate the instantaneous frequency in multi-component signals, Linschmann et al. (31) proposed two methods based on selective filtering and the Hilbert transform for obtaining the respiration from BCG signals.

Similarly to BCG, photoplethysmography (PPG) provides critical physiological information, including heart rate and blood pressure, in a non-invasive, easy-to-obtain, and cost-effective way, so as to be widely considered for deployment in home settings (32). Various attempts have been made to use PPG in combination either with BCG (33) or with ECG (34) by calculating the pulse arrival time (PAT) value.

**Figure 1:**
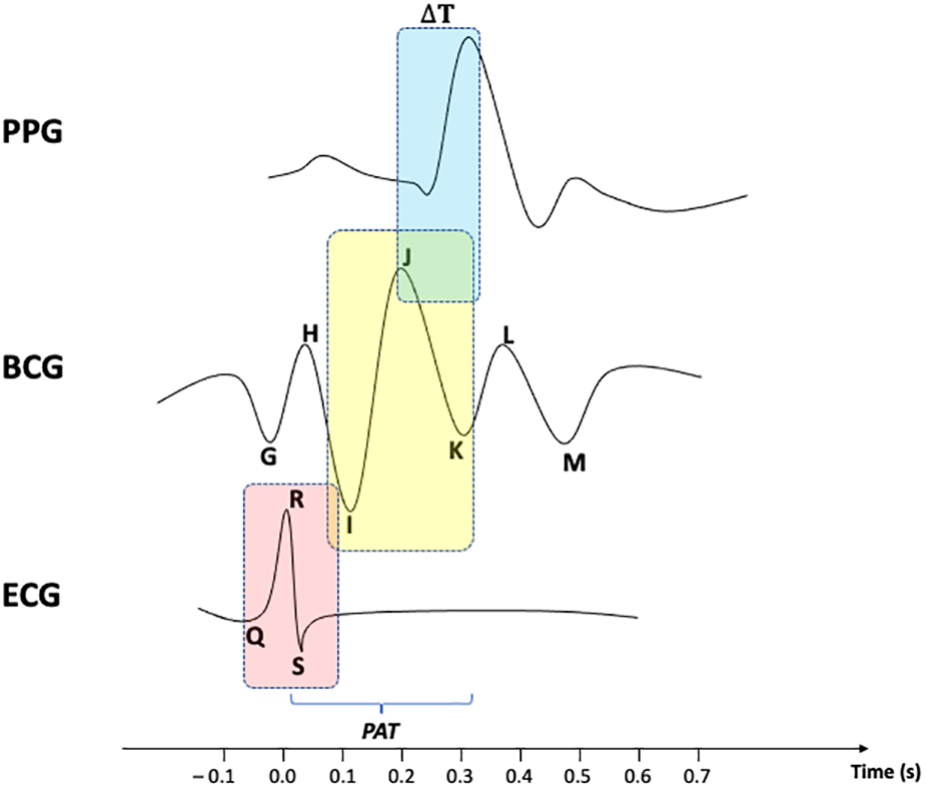
Comparison over time between BCG waveform, with ECG and PPG signals.

As shown in Figure 1, PAT is usually referred to as the total time delay between the R-peak of an ECG and a given feature point of a PPG. Since many scholars [see e.g. (35),] have provided reliable methods to evaluate the time delay between ECG's R-peak and BCG's J-peak as a measure to estimate systolic blood pressure (SBP), the remaining *Δ*T between BCG waveform and PPG signal can be accordingly identified as well. Nonetheless, noise and motion artifacts can corrupt PPG signals, thus making peak detection problematic. Bhowmik et al. (36) provided an algorithm for removing the baseline drift in the signal using wavelet filtering and trend removal for more reliable peak detection. The method was validated by comparing PPG peaks with RR series extracted from an ECG signal. In the work of Ferreira et al. (37), a prototype was described that makes use of an optical flow sensor to measure the relative displacement between a PPG sensor and the measurement site. The initiative showed a clear correlation between the motion recorded by the sensor and the artifacts contained in the PPG signal. The authors performed a principal component analysis for denoising the signal and a peak finding method to evaluate HR. More recently, a novel hybrid method was developed by Ahmed et al. (38), which leverages deep learning to denoise a PPG signal by decomposing it via a fast wavelet transform, and then reconstructing it by means of a custom feed-forward neural network. The mean squared error was computed between the obtained denoised sequence and the reference clean PPG signal to assess the feasibility of the approach.

### 2.2. Telemedicine solutions/architectures

The eHealth monitoring systems scenario is receiving a great deal of attention from healthcare operators, either academic or professionals. This is mostly due to the exponential improvement of the available digital technologies that, adding cutting-edge functionalities, allow the development on a great scale of those application models foreseen since the original definition of the eHealth paradigm at the beginning of the 21st century (39, 40).

Almost any of the debated works deal with the use of microcontrollers to gather data from the sensors. In these solutions, microcontrollers are usually coupled with patients' smartphones to act as gateways for the internet in order to send data to central servers. A process like this also implies several degrees of collaboration from the patient. For example, this is the case for a system where (i) data gathered from a self-developed sensor are sent to a Raspberry Pi as a data aggregator, and (ii) patients' smartphones are then used as a gateway to the internet (41). Other authors proposed a solution that, although based on Android and IoT, allows real-time gathered data transmission only through a patient's smartphone (42). Furthermore, Jenifer et al. (43) developed an IoT-based health monitoring system that makes use of a patient's smartphone connected to the home Wi-Fi to send the collected data to the cloud. Such systems often involve a pair of microcontrollers (one dedicated to sensor communication with raw data collection, and the other dedicated to the data aggregation and the subsequent forwarding to a central server), which makes the whole solution cumbersome and barely tolerated by the patients. In this regard, Hamim et al. (44) presented an IoT-based monitoring system solution that relies on two microcontrollers: an Arduino UNO and a Raspberry Pi 3. The former is used for sensor communication and the latter for network communication, while the cloud server hosts patients' data. The solution proposed instead by Al-Sheikh and Ameen (45) featured a NodeMCU that was used for communication with prototypal sensors for data collection, and an Arduino was used for data aggregation and data forwarding to a cloud service. An IoT–based healthcare monitoring system that encompasses several microcontrollers was deployed by Gupta et al. (46), where a NodeMCU was used for serving UI and interacting with the patient, an ESP8266 was in charge of interacting with the sensors, and an Arduino was used for data aggregation and forwarding to the central server.

In addition, sensors embedded in the monitoring system architecture are in many cases “homemade craft”, developed with the use of the corresponding microcontroller module. This approach makes the sensors' readings mostly inaccurate due to the nature of the sensor itself—more prototypal than properly engineered and ready for the market. Such modules are intended for being used while designing software architectures, so they need to be replaced with proper monitoring devices that embed the sensor corresponding to that module. In this way, they can be used with real patients in testing sessions. In this sense, Swaroop et al. (47) developed a monitoring system where the data collection is delegated to Raspberry sensor module DS18B20. Despite the good performances of the architecture as a whole, the authors themselves stated that the accuracy is limited by the sensor. A system based on the use of an ESP32 microcontroller was instead proposed by Manoj et al. (48). The set of sensors embedded was made by AD8232 and MAX30100 modules for ESP32. Also, in this case, the prototypal nature of the architecture somehow hindered the overall level of accuracy of the system.

The above-mentioned limitations may also reverberate on the lack of tolerability from the patients during the testing session, up to a complete uselessness for the proposed aim, as registered by Dhruba et al. (49) who deployed a solution for monitoring sleep apnea by embedding MAX30102 and AD8232 Arduino modules. The system was able to successfully detect sleep apnea in some cases, yet in most of the occurrences, patients reported that the worn devices would come off and cause a general uncomfortable feeling, therefore spoiling their ability to sleep. In this regard, BCG signal processing draws major attention from the scientific community for the continuous remote monitoring of the status of patients in unique conditions (e.g., sleeping time) due to its non-contact feature. Home monitoring of a patient's condition took advantage thanks to the ease of use of both modern BCG systems and related monitoring architectures, less obtrusive than those embedding contact-based devices, such as pulse oximeters, electrocardiographs, and smartwatches (8, 50). The same effectiveness was also proved during the COVID-19 pandemic (51). Different categories of BCG instrumentation have been used by the scientific community while trying to address in particular the problem of continuous and unobtrusive cardiac remote monitoring (52, 53). BCG signal also proved to be comparable with both PPG (54, 55) and ECG (56, 57) signals to have a ground truth value while checking HR estimation performances. Therefore, rising interest and novel instrumentation are bringing to the development of novel signal processing techniques focused on both BCG signal modeling (58) and features extraction—Deep Learning, see e.g. (59). Continued innovation in both instrumentation and signal processing regards are improving the usefulness of BCG as a clinical tool.

## 3. Materials

The RPMS proposed for the TM-related solution aims to overcome the aforementioned set of problems, by relying on (i) data collection devices, namely the sensors, already present on the market, capable of assuring tolerability for what concerns cumbersomeness, and (ii) Android-powered gateways that allow not only an all-at-once communication with sensors and central servers, but also an entirely unattended data collection process.

### 3.1. System components

**Figure 2:**
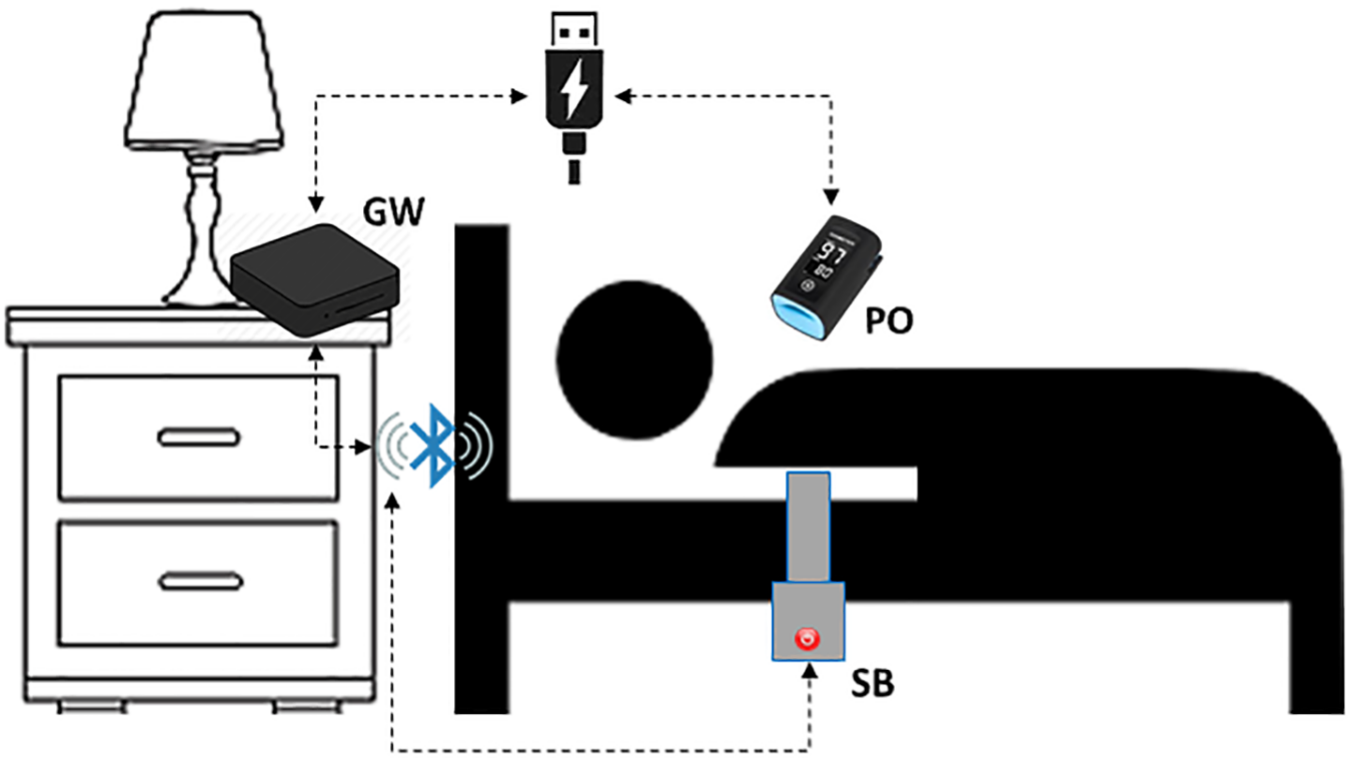
Measurement setup for the proposed architecture.

The proposed system relies on three different components (Figure 2):

• Sleep Belt (SB)
• Pulse Oximeter (PO)
• Gateway (GW)

The sleep belt used for the collection of the BCG signal in a non-invasive way is the J1657 model, manufactured by J-Style Joint Chinese Ltd. It is a low-cost non-wearable smart sleep monitor already available on the market. It consists of a pressure sensor band spanned along its entire length and is able to transmit a raw pressure signal at a 50 Hz frequency. The device is composed of a strap at the end of which a main body is placed. The strap's dimensions are 800 mm in length and 55 mm in width. The SB is designed to operate while placed under bed sheets, thus satisfying the non-contact constraint while not giving any hassle to the monitored subject. The main body features a 350 mAh lithium battery and a Bluetooth 4.0 connectivity module within the sizes of 70 mm in 70 mm. Making use of high-sensitivity sensors, the device produces signals specifically targeted at retrieving the subject's heartbeat, respiration rate, and movement. The device communicates with the gateway via Bluetooth protocol thanks to an ad-hoc application installed on the GW and developed thanks to the Software Developer Kit (SDK) supplied by the belt manufacturer. The SB is placed horizontally under the participant's bedding at chest height and is connected to power to avoid interruption of service. The sampling session starts automatically as soon as the device reads a continuous pressure, and thus data is broadcasted.

The pulse oximeter is used for collecting the PPG signal that will act as the gold standard. The finger-worn BM3000B USB Pulse Meter, by Shanghai Berry Electronic Technology Co., used for the trial is a low-cost device already available on the market. Its technical specifications declare ±2 bpm accuracy and 1 bpm resolution. Sensor measurement wavelengths are nominally 660 nm for red LED and 940 nm for infrared LED, while the maximum optical power is 4 mW. PO communicates with the GW via serial protocol by means of the application installed on the GW. This application was developed thanks to the SDK supplied by the PO's manufacturer. The device samples data at a 100 Hz frequency related to the oxygen saturation of the hemoglobin present in the arterial blood and the heart rate. This is possible thanks to the optical sensor that evaluates the reflection index of the blood flow through vessels. PO is connected to GW through a USB port that also provides power to the sensor. As soon as PO is connected, the sampling session starts and data flows through the serial channel.

The gateway is an Android Box featuring 4 GB RAM and 64 GB internal memory. It runs Android OS and is equipped with both a network interface (wireless and wired) and a Bluetooth interface. The GW hosts an *ad hoc* application developed in Java for Android. This is able to communicate with sensors to send commands for hardware control and receive data in real-time. Data transmitted from the sensors are collected at regular intervals and subsequently forwarded, via the Internet, to the remote server (RS) to be processed and displayed. The GW is connected to power and the wireless local area network (WLAN). When the system boots and the Internet connection is available, the developed application starts acquiring synchronous data from both the sensors embedded in the SB and the PO.

### 3.2. Architecture overview

**Figure 3:**
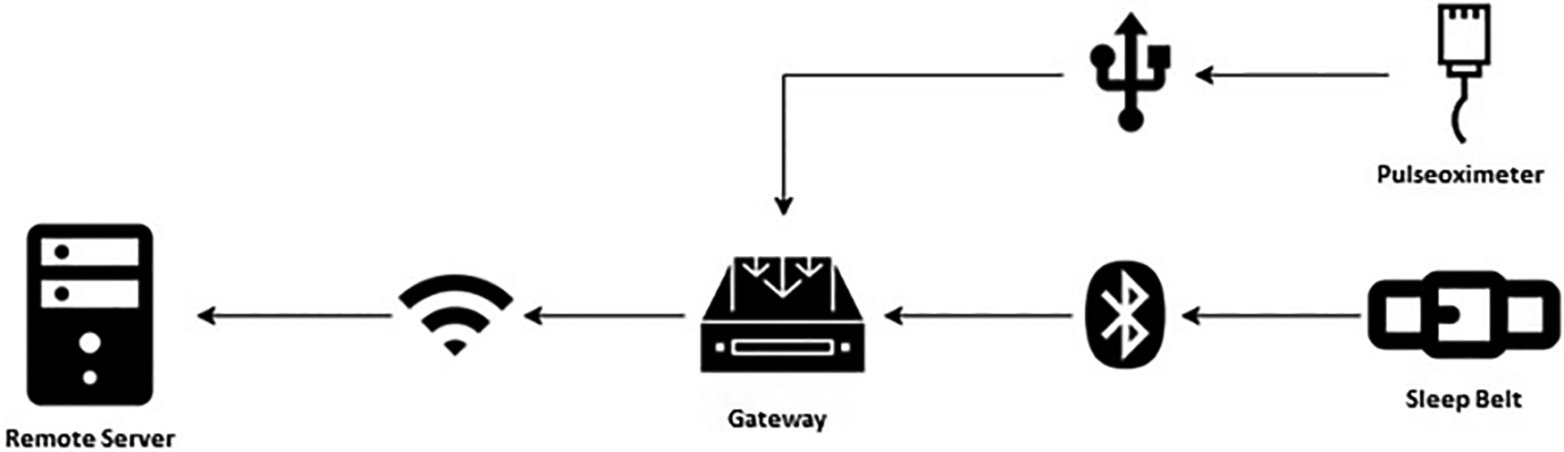
Architecture components and their connections.

The mentioned components interact within the architecture designed for signal acquisition, as illustrated in Figure 3.

The samples gathered in real-time from the monitoring devices (SB and PO) are divided into packets of equal size so that each data packet contains the same amount of information. Data packets are also equidistant from each other, thus generating time windows of interest of equal size. The data contained in each packet is framed in JSON files by the GW, before being transferred to the RS. The GW, whose time is synchronized by network time protocol (NTP), assigns the timestamp to the received data. The JSON files are transferred to the RS at regular intervals (1 min for PO, and 10 min for SB) after being marked with the time of transfer.

## 4. Methods

**Figure 4:**
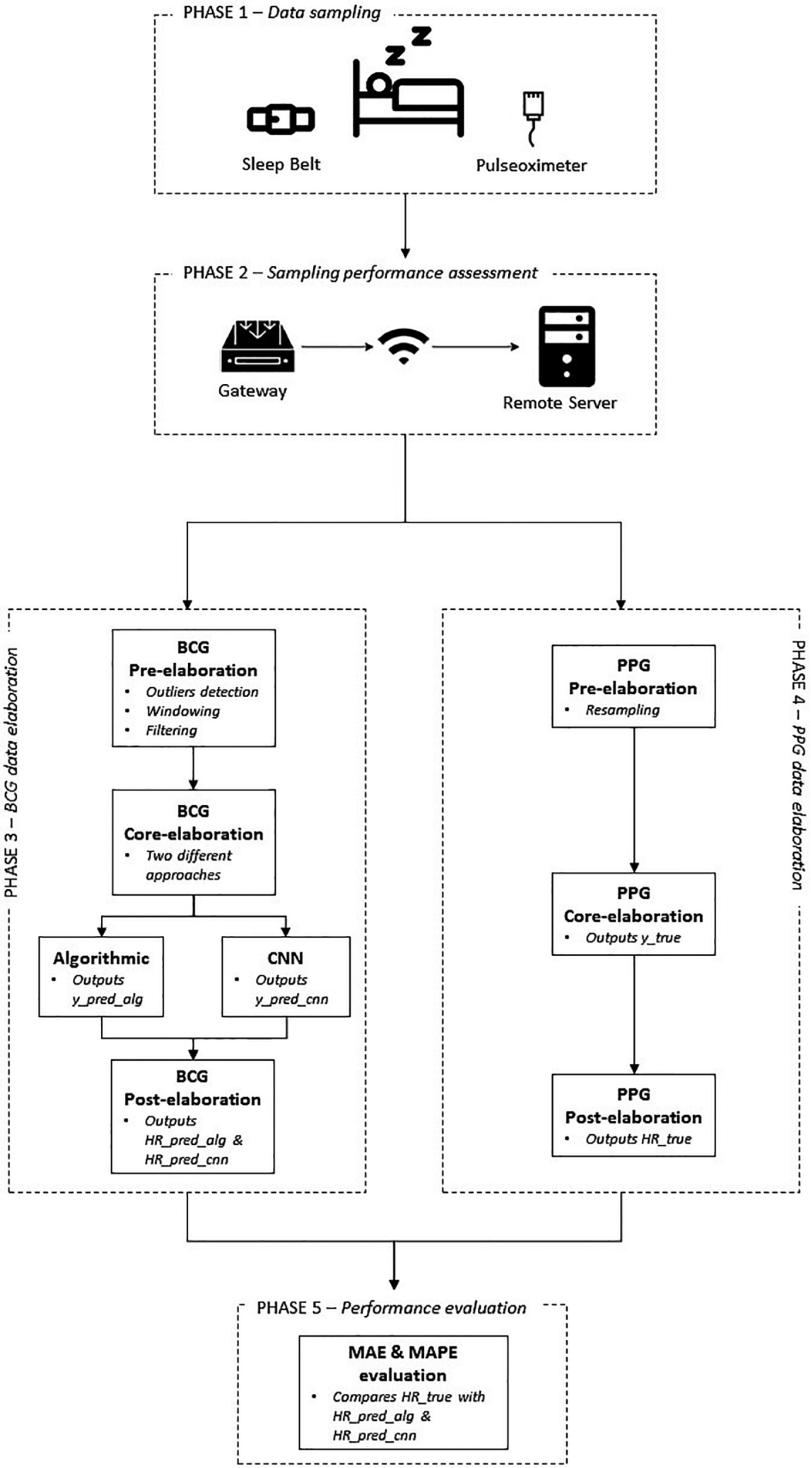
Methodology workflow.

Figure 4 depicts the workflow describing the main phases the field trial was organized into.

### 4.1. Phase 1—data sampling

The field trial was conducted in a non–medical context during the period September/November 2022 in a cohort of 24 volunteers, 14 females and 10 males, differing in weight (72 ± 18 kg), age (46 ± 19 years), and height (175 ± 10 cm) and who were not patients of a healthcare organization.

The main scope was to validate the correct functioning of the proposed architecture for the continuous remote monitoring of the participants' status during sleeping times. To this purpose, it was necessary to assess the data collected from the architecture — the BCG signal — and then elaborated via the approaches outlined to get results as to the HR extracted for each subject, in terms of robustness and consistency. Volunteers were asked to lie down on a sleep belt, which had been placed on the mattress and produced a BCG signal during nighttime, and to sleep for one night with the belt placed under their bedding. They were not asked to change their normal sleep habits. Each volunteer also agreed to wear a finger pulse oximeter for the entire duration of the sleep period, in order to provide a PPG signal as the gold standard for the heartbeat.

### 4.2. Phase 2—sampling performance assessment

To assess the performances of the communication between sampling devices (Pulse Oximeter and Sleep Belt) and gateways within the developed prototypal architecture, the packet loss — i.e., the ratio of data packets correctly transferred from the devices to the gateway — was evaluated (60–62).

### 4.3. Phase 3—BCG data elaboration

In the following sub-paragraphs, the approaches adopted for BCG data elaboration are described in detail.

#### 4.3.1. BCG signal pre-elaboration

BCG is generated from the body's vibrations due to the heart pumping blood to vessels. This means that the BCG signal can be easily contaminated by other vibrations, such as respiration, talking, and body movements. Other common sources of noise are power line interference and muscle contraction. The effective frequency of BCG lies in a low-frequency band.

During the sampling sessions, the entire length of the BCG signal generated from the sleep belt was split in windows of 400 samples each (*windowing*), corresponding to 8-second length intervals, which for a healthy subject in conditions of rest normally feature between 5 and 8 heartbeats (8, 63). References to different durations for the fragment length of the BCG signal can also be retrieved from the literature, e.g., 20-second segment fragments in Huang et al. (64), 30-second records in Cathelain et al. (65), 10 s in Vijayarangan et al. (66), and 1 s (91 samples at 100 Hz) in Hai et al. (67). In Malali et al. (68) 2.8-second windows of the ECG signal, sampled at 250 Hz, were used for segmentation. The choice is motivated by the specific technique, the testbed settings, and the length of the full registrations. In the present work, 8-second windows were set up to analyze the obtained results in light of what was reported by Pröll et al. (18).

**Figure 5:**
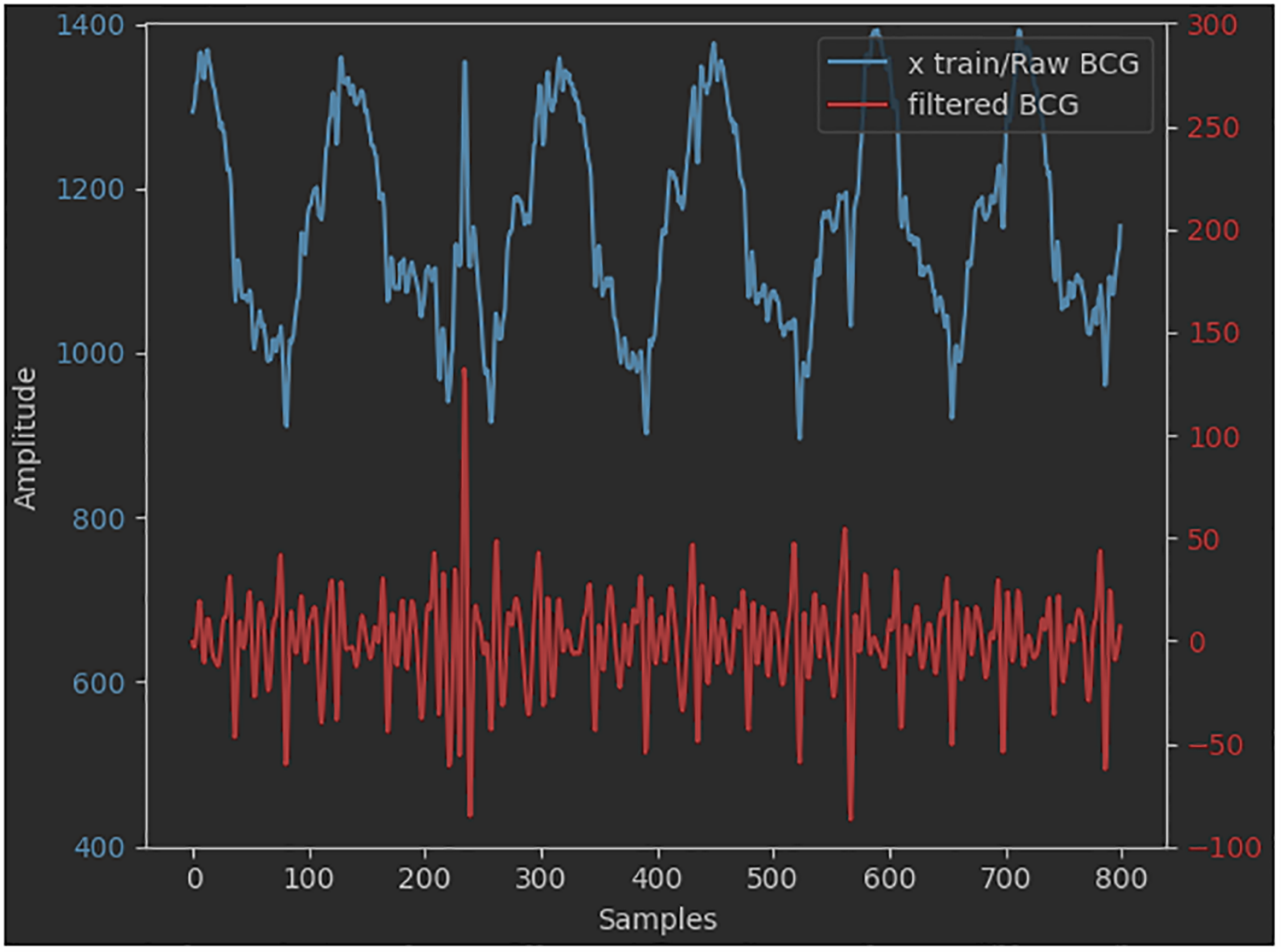
Raw vs. filtered BCG signals.

A pre-elaboration step was performed to shift from a raw signal to a filtered one. It consists of the detection and deletion of eventual outliers. A further operation is the modulation of the saturation values of the belt's pressure sensor, due to, for example, episodes of sudden movements of the subject, to generate a time series of plausible values. In this regard, the deleted values are usually replaced with contiguous ones to keep the overall trend of the series itself as seamless as possible. Many approaches use coarse low-pass filters to pre-process the original BCG signal. For example, high-pass filters (0.2–0.4 Hz) are used to remove the baseline signal and in particular the respiratory component (67). Low-pass filters —20 Hz (15, 16) or 30 Hz (17) —are used to remove fluctuation signals. In our case, pre-elaboration is a common step for two subsequent core-elaboration approaches, one of which is deep learning-based. Therefore, similarly to what was reported by Pröll et al. (18), Hai et al. (67), and Huang et al. (64), the original amplitude of the signal was reduced (*normalization*), by means of a narrower second-order digital Butterworth passband filter (2–10 Hz and 3 dB cutoff frequency) since no other filters were applied before the CNN training phase. The range of values chosen for signal filtering corresponds to the frequency interval within which the dominant waves of the BCG signal are comprised. This is also evident from the results of the FFT applied to the signal (69, 70). A comparison between the raw and the filtered BCG signals is shown in Figure 5.

The output of the initial signal processing step was then analyzed by means of two approaches, one algorithmic and one CNN-based.

#### 4.3.2. BCG signal core elaboration via algorithmic approach

**Figure 6:**
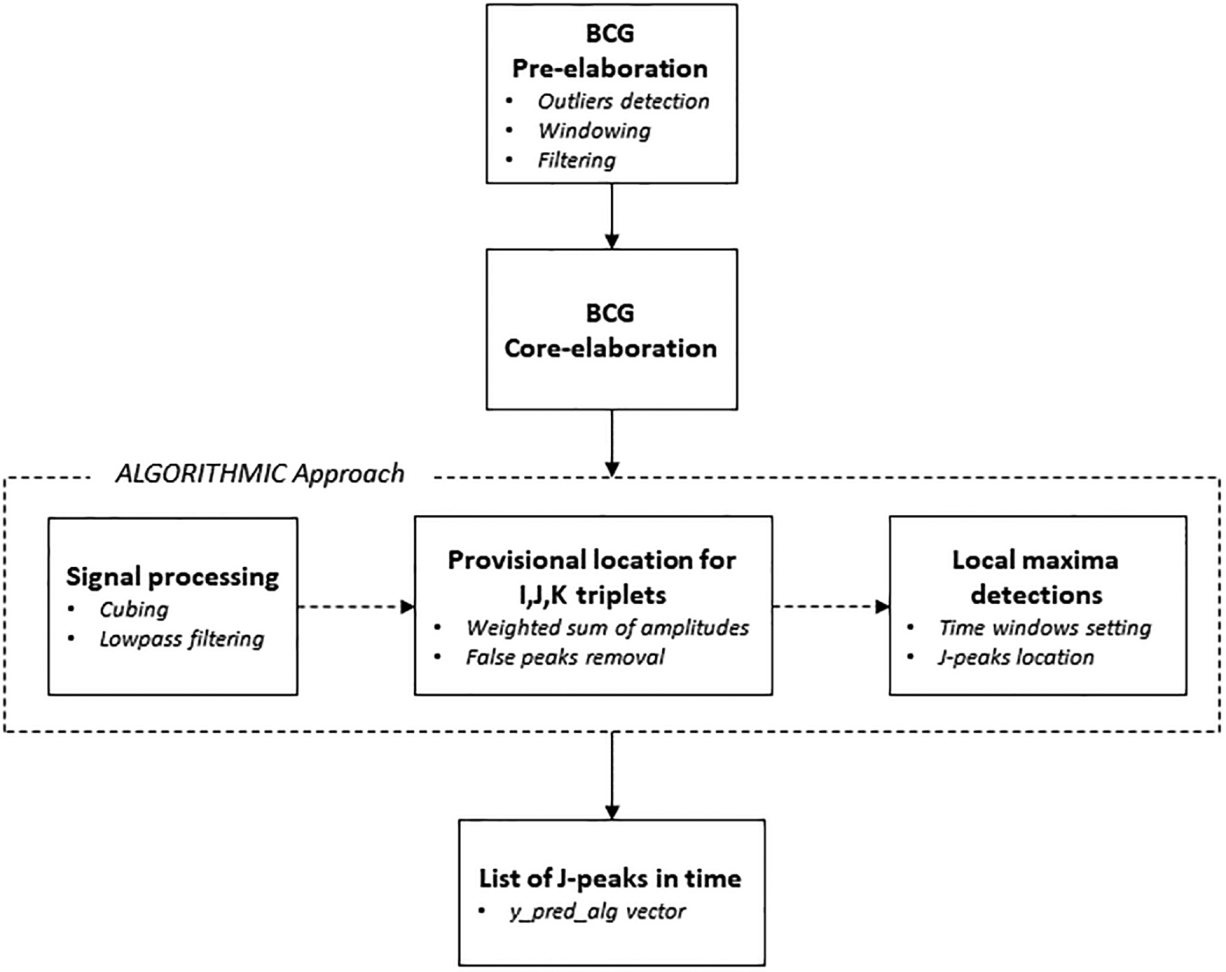
Main phases of the algorithmic approach.

The main reference from the literature was the segmentation algorithm approach introduced by Pröll et al. (18) that combined and extended aspects of several previous algorithms, assessing the overall performance by using the detected J-peaks for heartbeat estimation (71, 72). In the developed approach, several signal processing steps were performed (see Figure 6). The first step consisted of the enhancement of the ejection waves by conducting a cubing (*x*^3^) operation on the original BCG signal while keeping the signal sign intact. Following, the cubed BCG signal was lowpass-filtered, so as to be used to estimate the coarse position of the IJK complexes. Starting from this and considering the typical shape of the systolic complex, it was possible to determine the exact locations of the I, J, and K deflections, in order to get to the first list of local maxima (73). It can be supposed in fact that an IJK complex is composed of three consecutive local maxima (triplet), corresponding to a valley-peak-valley sequence in the processed signal. For each triplet, a weighted sum of the deflection amplitudes was calculated at the corresponding positions in the coarse signal. The weights were set as follows: *W*_I_ = *W*_K_ = −1 and *W*_J_ = 1 for valleys and peaks, respectively. The triplet with the highest weighted sum was then chosen as the provisional location for I, J, and K. The so-called “false peaks” were then removed, meaning values too close to each other to feature as useful points for determining any IJK complex. To do this, a window was set whose length is equal to the heartbeat interval — usually 1 s, during which 4–5 peaks occur — and the local maximum was detected as the final location of the J-peak. The result was the vector (*y_pred_alg*) comprising the indices of the positions of the J-peaks calculated, that is, the heartbeats.

In the following post-elaboration step, the vector of predicted HR values (*HR_pred_alg*) was eventually calculated from the mean distance between J-peaks in the 8-second windows obtained from the BCG signal of the sample sessions.

#### 4.3.3. BCG signal core elaboration via the deep learning approach

**Figure 7:**
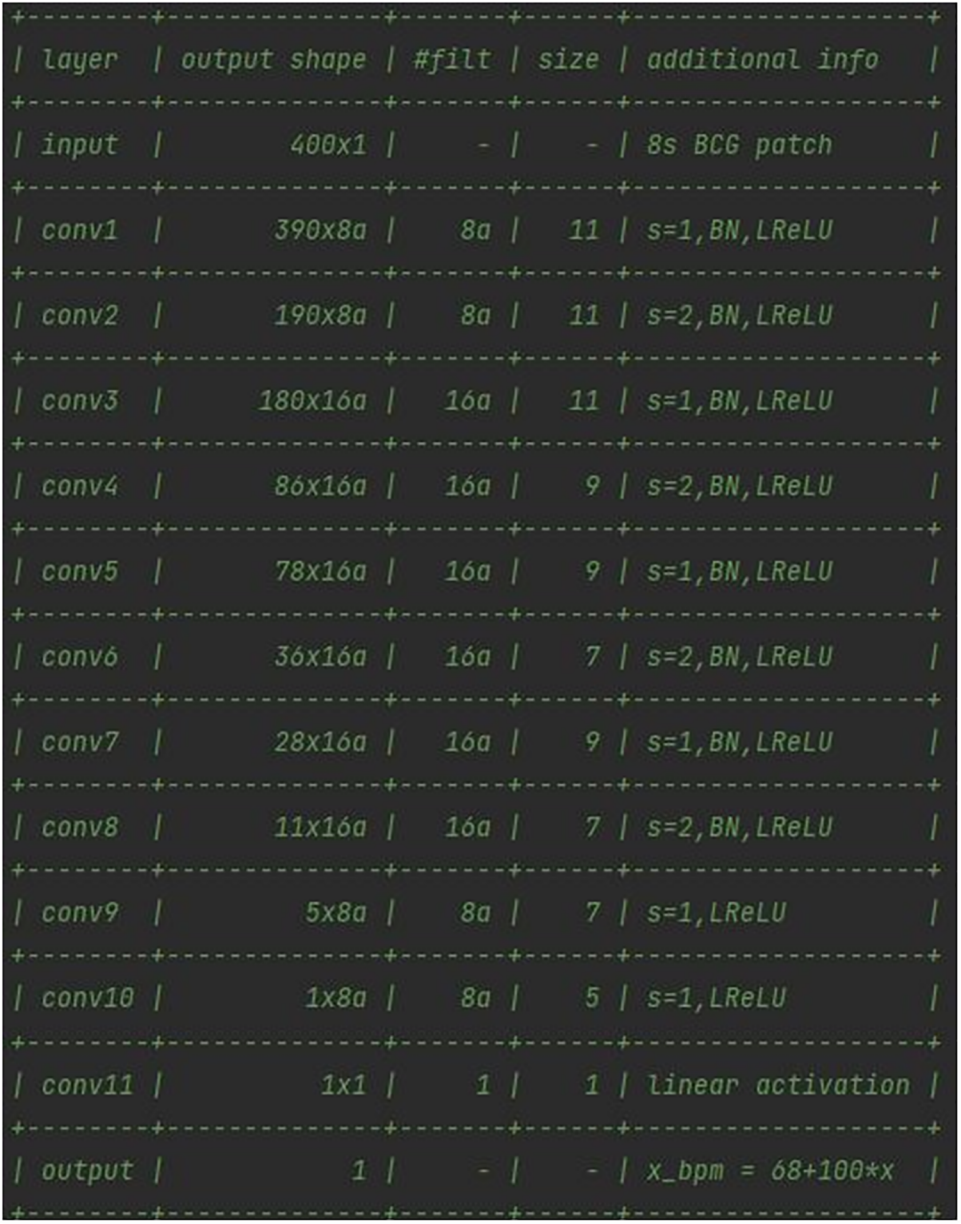
Architecture of the 11-layers CNN developed for the BCG signal elaboration.

In this case, starting from the analysis of the models of neural networks developed by Pröll et al. (19), an 11-layer convolutional neural network was figured out, which uses strided convolutions instead of pooling layers and does not feature any fully connected (dense) layers in the final stage (74). The CNN was optimized via the Adam optimizer (75) using default parameters, with Keras as a kernel regularizer. As reported in Figure 7, every convolutional layer of the network, except the final one, was followed by a batch normalization (BN) and a leaky rectified linear unit (ReLU) layer.

On the one hand, BN was deployed as a sole measure to provide some form of regularization, and to improve training behavior (76, 77). On the other hand, leaky ReLUs can reportedly improve performance, although only to a small extent (78). The final layer used a single filter of kernel size 1 to obtain a single value from the previous feature channels. Target HRs were normalized to mean = 0 and variance = 1, based on heart rates in the training set. The loss function chosen was the mean squared error between normalized HR and network output. In a series of 11 convolutional layers, the input patches of size 400 (8 s at 50 Hz sampling rate) were reduced to a single output value.

**Figure 8:**
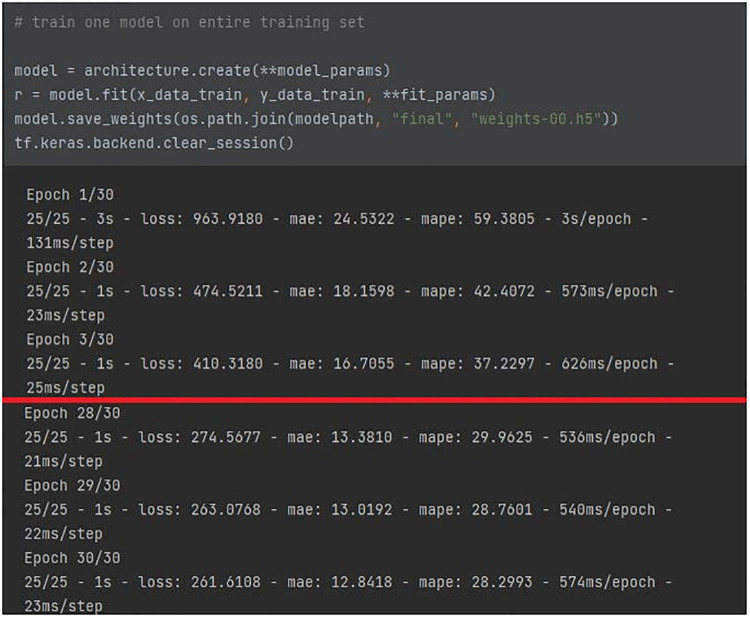
CNN training over epochs.

The deep learning model was implemented by means of an HPC server equipped with an NVIDIA GPU for the data center and AI with 48 GB of VRAM, and allocated at the IBB-CNR research facility. The full architecture of the network, reported in Figure 7, featured 18.8 k parameters. The *a* means that the number of filters can be increased by a constant factor for all layers. Given the high number of parameters, it was chosen to keep the factor *a* = 1, in order to prevent an enlargement of the original model of the network from affecting the computational load requested. The training population consisted of 16 volunteers (67% of the total participants involved) who were randomly selected prior to any network optimization. The BCG signal of the training population, split into 8-second windows, was normalized and used as inputs for the network. The CNN was trained for 30 epochs, as the training error converged after around 15 epochs. No pre-processing or data augmentation were used for training. No early stopping was implemented and the final model state was used for evaluating performance, as illustrated in Figure 8.

The red line traced in the figure shows the improvement of the architecture parameters from the first to the last epoch: a halving is evident for either MAE/MAPE, and the loss function. The performance was additionally supported by leave-one-out cross-validation on the training set. The ratio of testing data was 33% of the original data set (equal to 8 participants, randomly selected prior to any network optimization). Since no real hyperparameter tuning was employed, there was no need for an additional subdivision to obtain a validation set.

The output of the network was the vector (*y_pred_cnn*) comprising the indices of the positions of the real J-peaks over time, that is, the heartbeats. A further post-elaboration step allowed the calculation of the vector of predicted HR values (*HR_pred_cnn*), calculated from the mean distance between J-peaks in the 8-second input windows, as seen in the algorithmic approach.

### 4.4. Phase 4—PPG data elaboration

**Figure 9:**
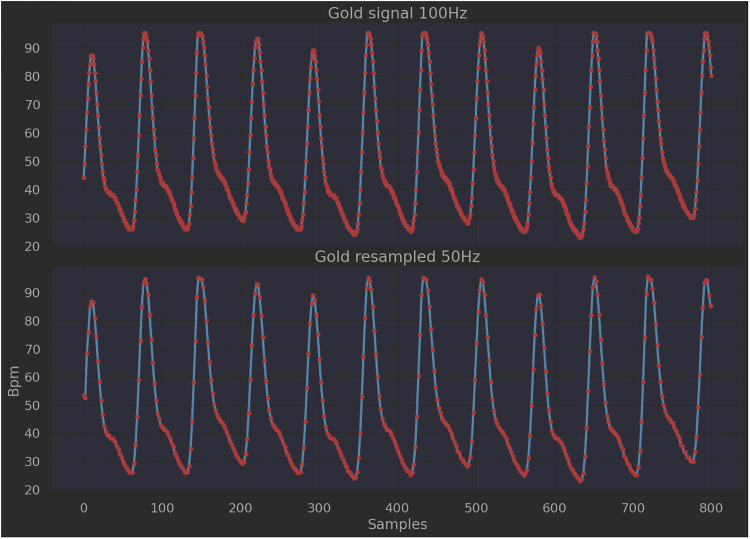
PPG signal before and after resampling.

To assess the performances of the two approaches, the PPG signal collected from the finger pulse oximeter was used as the gold standard, since the device produces a clean signal whose pattern can be compared with the BCG filtered one. As shown in Figure 9, it was necessary to perform a resampling on the PPG signal (pre-elaboration step), as the working frequency of the pulse oximeter (100 Hz) is different from that of the sleep belt (50 Hz). The number of samples considered per time unit was therefore decreased through linear interpolation operations. Following this, the PPG signal was also split into 8-second length intervals (*windowing*).

In the core-elaboration step, the locations of the peaks generated from the heartbeat were searched within the aforementioned 8-second length intervals using the method of local maxima and minima (79). The output was the *y_true* vector that features the heartbeat locations over time. The true heart rate (*HR_true*) vector from the PPG signal was eventually determined in the post-elaboration step, by means of the following equation:

$$HR=\frac{60\;*\;F_{s}}{N_{s}}$$

where *F_s_* means the sampling frequency, and *N_s_* means the index point corresponding to the peak location (80).

### 4.5. Phase 5—performance evaluation

The performance checking was evaluated in terms of patient-wise MAE (mean absolute error) and MAPE (mean absolute percentage error) between the *HR_pred* vectors from the BCG signal-related approaches, and the *HR_true* vector from the gold standard (PPG signal), since these metrics provide valuable insights as to inter-patient differences in waveforms and signal quality (see the following equations):

$$\mathrm{MAE}=\frac{1}{m}\overset{m}{\underset{t=1}{\sum }}|e_{t}|$$

$$\mathrm{MAPE}=\frac{1}{m}\overset{m}{\underset{t=1}{\sum }}\frac{\left|e_{t}\right|}{y_{t}}$$

$$e_{t}=HR\_pred_{t}-HR\_true_{t}$$

$$y_{t}=HR\_true_{t}$$

where *m* is the number of intervals considered.

## 5. Results

It emerged from the trial stage that the average length of signal collected during the sleep time was 7 h and 12 min, with a standard deviation of 30 min. Such signal length was not always entirely analyzable due to the signal saturation introduced by the nature of the sensor itself (81). It was noticed that saturation occurred when the subjects tossed or moved during sleep. Such events affected 15% to 20% of the signal length on average, so no less than 80% of the signal was available for elaboration. In addition, in the tested architecture, a 10% average packet loss ratio was found. This means that not all the data packets collected by the sleep belts were intercepted by the gateway. In order to find out the origin of the issue, first, an analysis of the transmitted data packets was performed by means of a dedicated computer sniffing packets leaving the belt. The resulting packet loss ratio, related to the comparison between packets sniffed and belt sampling rate, was 0.5%. An ad-hoc application was then installed on the same device that would act as a gateway in the architecture, to receive belt data packets. In this case, the result was a 14% packet loss ratio at 100 Hz and 8% at 50 Hz. This is probably because the GW is periodically unable to consume the Bluetooth data queue.

Further, very different elaboration times were observed between the two proposed approaches for the BCG signal core elaboration. In particular, the process execution time of the algorithmic approach —which is by nature purely sequential — exceeded 5 h per patient per night (315 min ca.), with a resulting ratio between elaboration time and signal length of about 70%. Elaboration time stayed high although several precautions were followed during algorithm implementation, such as break conditions, variables releasing, and memory cleaning. The deployment of a CNN-based approach allowed for the elaboration time to dramatically decrease. After the proper network training, in fact, it resulted in about 35 min per patient per night during the testing phase, with a resulting ratio between elaboration time and signal length of about 0.7%.

Table 1 lists the patient-wise average and standard deviation of MAE, comparing the two approaches performed by Pröll et al. (18) and Pröll et al. (19), with what has been described in the present article for a TM-related environment.

**Table 1:** Patient-wise MAE comparing Pröll's and the authors’ approaches (in bpm).

Source	Model	Avg.	Std.	Params.
Pröll et al., (18)	Algorithm	4.24	2.21	—
Authors	Algorithm	5.46	3.31	—
Pröll et al., (19)	Modified CNN-GRU × 0.5	2.32	1.14	8.3k
Authors	CNN	3.54	2.11	18.8k

Pröll's approaches delivered an average MAE of 4.24 and 2.32, respectively. Both these values outperform those resulting from the field trials conducted by the authors (5.46 and 3.54) by 29% and 52% ca., respectively. For the standard deviation, the data collected and elaborated by the authors via the algorithmic approach delivered a value that exceeds that achieved by the corresponding model of Pröll *et al*. by about 50%. A more significant percentage difference (85% ca.) occurred between the two CNN-based approaches. Further insights come from the evaluation of MAPE, which was 9.1% (std = 0.77) and 6.3% (std = 0.48) for the algorithmic model and the neural network, respectively.

A comparison was also conducted with high-complexity reviewed methods for processing PPG signals extracted from wearable sensors to estimate HR values (82, 83). For instance, a general framework, termed TROIKA, was proposed by Zhang et al. (84). Experimental results on datasets recorded from 12 participants using smartwatches showed that the MAE of heart rate estimation was 2.34 bpm. Starting from the TROIKA dataset, Biswas et al. (85) deployed a four-layer deep neural network on 22 PPG records collected during various physical activities, achieving an MAE of 1.47 bpm. Also noteworthy is the experimentation carried out by Arunkumar and Bhaskar (86) who developed a de-noising algorithm for reducing the impact of motion artifacts during HR estimation using a phase vocoder. The test on 22 datasets resulted in an MAE of 1.86 bpm.

## 6. Discussion

The major challenges regarding the analysis of BCG signals are the inevitable capture of other forces produced by the human body and the measures being highly dependent on the setup and placement of the sensors, not to mention body movements. Moreover, such issues are amplified when it comes to a non-controlled environment. In this regard, the main reason behind the gap between the results, as shown in Table 1, lies in the original BCG signal, which in turn depends on the dynamics concerning data collection. Due to the peculiar characteristics of any TM-related environment and the low cost and low invasiveness of the proposed architecture, the collected BCG signal stands on a different order of magnitude from those treated in Pröll et al. (18) and Pröll et al. (19). This causes the signal impurities and variations to be amplified as well, transferring such uncertainty from signal sampling to the following elaboration steps. Additionally, during the study, the signal was collected continuously all night long. Subjects were not asked to change their normal sleep habits and to set up a scenario as closely related to reality as possible. Eventually, they were advised that in the case of any inconvenience, the sampling session for that night would be stopped.

The analysis of the achieved ratios between the BCG signal length collected during the trial, and the two signal elaboration times, made it clear that the algorithmic approach is not preferable in TM-related environments, as the amount of signal data to be elaborated demands high machine-time involved in data processing and, accordingly, an elevated computational cost. This is a strong limitation because the huge amount of data coming from remote information, signals, and image transmission that telemedicine architectures deal with is fairly comparable to many other big data-related scenarios (87). In addition, the algorithmic approach was outperformed by CNN during core elaboration in terms of both time and cost. In this regard, for the CNN design, we used the Modified CNN-GRU × 0.5 as the reference model among those developed by Pröll et al. (19), as it was the best result in terms of MAE avg vs. parameters. Our network featured more convolutional layers with smaller kernels to achieve better accuracy at a similar network size. No recurrent layers were introduced as it was critical for us to pursue an optimal balance between the number of filters per layer (which improves the estimation accuracy) and the number of resulting parameters (which would cause a computational load that was too high). This is because, in a TM-related environment, a lightweight architecture is always a condition of utmost importance to fulfill, in order to keep competitive performances even with huge amounts of data to handle. Such an aspect is related to an elevated (i) number of participants to monitor; (ii) monitoring timespan, and (iii) time length of the data collected. In other words, any proper telemedicine initiative is meant by nature to be both widespread and continuous over time (many hours per day, for many days). For the data packet loss ratio, this could be probably be enhanced by working on the data receiving buffer, either on the application side or on the Bluetooth system's library side so as to optimize the GW software. Modifying buffer size and access could likely lead to better packet loss values, yet it was decided not to move in this direction to evaluate the real gain. The reason for this choice was that the signal gathered was considered good enough for fair signal processing. Further, modifying the system's libraries to allow the GW to consistently reduce packet loss, as beneficial as it might be, would have been too much of a time-consuming task. This is not to mention that more powerful hardware, which on the one hand may lead to better performance, on the other hand, would entail higher costs.

This proves that the prototypal architecture proposed is capable of addressing both the issues of low cost and low invasiveness. The percentage of the available collected signal was revealed to be sufficient to perform a robust data elaboration. This implies that the device chosen for data collection, although low-cost and already on the market, is suitable for the intended purpose.

Many other scholars have proposed novel solutions for the assessment of HR from the sampling of the BCG signal in TM-related environments. For example, Dozee, introduced by Saran et al. (88) is a contactless vital parameter monitoring system designed to measure heart rate and respiratory rate continuously and without contact in a hospital setting or at home. The authors deployed a unique unsupervised clustering algorithm that proved to effectively isolate cardiac contractions and respiratory events from the unconstrained BCG signal. Aydemir et al. (89) analyzed BCG signals collected in the home environment for remote monitoring of heart failure (HF) via a set of sensing approaches, comprising a modified weighing scale, a wearable accelerometer, a wearable camera, and a toilet seat. A LOSO (Leave One Subject Out) cross-validation was implemented to predict the generalization performance of the classifier in all experiments, in order to establish the feasibility of HF status classification and, therefore, to investigate the prediction of future decompensation risk. In this case, despite the capacity of the system to integrate well with participants' IADLs, the need for active participation from the user to enable many of the mentioned devices still makes the effectiveness of the approach questionable. An inflatable air mattress, capable of supporting up to 150 kg of weight, and integrated with an air pressure sensor as the only device needed to collect the necessary information, was instead the focus of the research conducted by Lin et al. (90). The low-cost and non-invasive system uses ultrasonic signals to detect the participant's turning movements. Traditional linear regression was used to model the relationship between the ground-truth signal and the explanatory (independent) variables of BCG heart rate, breathing rate, and sleeping position. Other non-contact, sensor-related technologies for the extraction of the heart rate from the BCG signal have only been tested so far in controlled environments — usually hospitals — although the final goal remains the achievement of effective household HRV monitoring. It is worth mentioning the solution proposed by Zhao et al. (91) who described a highly-sensitive fiber optic sensor for accurate and steady BCG signal detection, for which a deep learning-based method was proposed to improve the accuracy of the beat-to-beat interval (BBI) extraction from HRV analysis.

These studies show that this line of research features aspects related to technological process innovation. Further developments are therefore to be expected, either the creation/use of new technologies or the improvement of quality and efficiency of internal and external processes, to render services to users and citizens (92–94). More specifically, pursuing innovation means, in the present case, the joint evolution of organizational models, healthcare purposes, and technological components. In this regard, another critical perspective the authors accounted for focuses on the effort to develop technologies capable of guaranteeing an as comfortable as possible experience for the subjects involved. Results achieved in the study align well with the three aforementioned dimensions: (i) the deployment of non-contact, non-intrusive solutions allowed for participant's sleep habits overnight to remain unaltered (healthcare); (ii) the effectiveness of the experimentation also relied on the ease of use (plug and play) of the technological components (technology); (iii) the implemented system proved to be capable of the continuous remote monitoring of biomedical parameters, thus preventing the participants needing to refer to hospital-based environments (organizational model). An improvement was then realized under a sort of “3Ps” vision: patient-wise, person-wise, and subject (em)Power(ment)-wise, respectively (95).

## 7. Conclusions

In the present article, a low-cost solution for non-invasive remote monitoring of a patient's status during sleeping times was described. An analysis of the BCG signal for HR detection was performed. An RPMS was developed, which allowed us to address critical issues related to (i) the use of unattended and unobtrusive devices for data gathering, (ii) the possibility to spread the solution to a wide cohort of participants, and (iii) the integration with participants' IADLs. In order to verify the reliability and applicability of the proposed system, the data collected were analyzed by means of two approaches —one algorithmic and one CNN-based — chosen after a thorough analysis of the literature. The quality of the results achieved, expressed in terms of MAE (3.54 bpm for CNN-based approach), was lower than those obtained in well-controlled environments [2.32 bpm, see (19)] and when relying on signals of different nature, such as PPG [1.47 bpm, see (85)]. Despite this, the architecture performance and the data elaboration approaches still show satisfying results. This, on the one hand, substantiates the reliability and applicability of the system, and, on the other hand, suggests room for improvement in both the methodologies of BCG signal processing and data analysis (96).

Steps forward are planned to further improve the solution. As the robustness of the architecture was certified, updates are currently being tested to implement more refined models where, for example, the data, once collected from the device, are soon transferred to the central server for real-time elaboration (97). The possibility of handling a greater computational load will likely allow research to explore new solutions in terms of (i) data analysis, and (ii) the design of more powerful models of neural networks featuring both convolutional and recurrent layers (98). This will make it possible to fulfill new targets, such as the evaluation of respiratory rate or pattern assessment from participant's movements overnight.

## Data Availability

The raw data supporting the conclusions of this article will be made available by the authors, without undue reservation.
